# Alcohol consumption during COVID-19 pandemic: What have we learnt so far?

**DOI:** 10.1192/j.eurpsy.2021.298

**Published:** 2021-08-13

**Authors:** V. Nogueira, M. Mendes, I. Pereira, J. Teixeira

**Affiliations:** Clínica 4 - Unidade De Alcoologia E Novas Dependências, Centro Hospitalar Psiquiátrico de Lisboa, Lisboa, Portugal

**Keywords:** COVID-19, pandemics, alcohol consumption, Psychological Distress

## Abstract

**Introduction:**

The current SARS-CoV-2 pandemic has many implications, one of them being alcohol consumption. The impact of long-term distancing measures in terms of alcohol use and misuse is yet unknown. Any increase, would not only add to the usual disease burden associated with alcohol, but also add to the COVID-19 load, given that alcohol use may weaken the immune response.

**Objectives:**

To characterize and compare the pattern of alcohol consumption throughout the pandemic in patients with the diagnosis of Alcohol Use Disorder; to identify factors considered as most relevant in the increase of alcohol consumption.

**Methods:**

We conducted a observational study in an outpatient population in Centro Hospitalar Psiquiátrico de Lisboa (Portugal) with diagnosis of Alcohol Use Disorder, 6 months after the pandemic lockdown. We characterized our sample regarding social, demographic and clinical categories. We applied auto-filled questionnaires, particularly: Mental Health Inventory (MHI), Positive Mental Health Scale (PMHS) and Severity of Alcohol Dependence Questionnaire (SADQ-C).

**Results:**

A total of 65 patients were included. More than 30% changed their drinking habits because of the pandemic. Nearly half of these increased consumption, and half decreased (16% vs 14%). The increase affected particularly men, and was related with the severity of alcohol dependence, stress-related coping strategies and psycopathology; on the other hand, a lowered level of consumption based on the decrease of alcohol accessability and affordability.

**Conclusions:**

The current situation is unique in terms of mass physical distancing and may trigger different behaviours that should be monitored. Governments should give public health warning about excessive alcohol consumption to protect vulnerable individuals.

**Disclosure:**

No significant relationships.

